# Primary Retroperitoneal Mature Cystic Teratoma in an Adult Diagnosed Using Percutaneous Needle Biopsy: A Case Report

**DOI:** 10.7759/cureus.107688

**Published:** 2026-04-25

**Authors:** Hideki Tsukada, Takayuki Hirano, Hiroyuki Hayashi, Shusei Fusayasu, Junichi Ohta

**Affiliations:** 1 Urology, Yokohama Municipal Citizen's Hospital, Yokohama, JPN; 2 Pathology, Yokohama Municipal Citizen's Hospital, Yokohama, JPN

**Keywords:** adult, extragonadal germ cell tumors, mature cystic teratoma, percutaneous biopsy, retroperitoneal tumor

## Abstract

Retroperitoneal teratomas are rare in adults and often pose diagnostic challenges when evaluated using imaging alone. We report the case of a 35-year-old man with a 10-cm retroperitoneal mass located adjacent to the upper pole of the right kidney, which was incidentally detected during a routine health checkup. CT revealed a fat-containing lesion, initially suggesting a renal angiomyolipoma. However, MRI demonstrated heterogeneous components with internal septations and calcifications, raising suspicion for other retroperitoneal tumors, including a liposarcoma.

Because imaging findings were inconclusive, an ultrasound-guided percutaneous core needle biopsy was performed. Histopathological examination revealed keratinizing squamous epithelium, skin appendages, adipose tissue, and smooth muscle tissue, findings consistent with a mature cystic teratoma. The tumor was surgically resected en bloc with the right kidney due to strong adhesion to the renal upper pole. Histopathological analysis confirmed a mature cystic teratoma without malignant components. This report highlights the clinical utility and novelty of percutaneous biopsy in establishing a preoperative diagnosis and facilitating appropriate surgical planning for complex retroperitoneal masses.

## Introduction

Teratomas are germ cell tumors composed of tissues derived from the three germ layers: ectoderm, mesoderm, and endoderm. They most commonly arise in the gonads, including the ovaries and testes, but can also occur in extragonadal locations such as the anterior mediastinum, retroperitoneum, sacrococcygeal region, and pineal gland [1]. Retroperitoneal teratomas account for approximately 1-10% of primary retroperitoneal tumors and are most frequently diagnosed during infancy and childhood [1]. In adults, primary retroperitoneal teratomas are rare [2], and mature teratoma is therefore not always considered in the differential diagnosis of retroperitoneal masses in this population. Patients with retroperitoneal teratomas are often asymptomatic, and the tumors are frequently detected incidentally [1]. Consequently, preoperative diagnosis of retroperitoneal teratomas in adults can be challenging due to their heterogeneous imaging features and overlap with other retroperitoneal tumors. We report a rare case of an adult retroperitoneal mature cystic teratoma located in the perirenal region that was diagnosed preoperatively using percutaneous needle biopsy.

Written informed consent was obtained from the patient for publication of this case report and any accompanying images. This study was conducted in accordance with the ethical standards of our institutional policies. According to our institutional guidelines, formal IRB approval is not required for a single case report.

## Case presentation

A 35-year-old man was referred to our department after a mass was incidentally detected during abdominal ultrasonography performed as part of a routine health checkup. The patient remained asymptomatic throughout the clinical course, with no onset of abdominal pain or systemic symptoms. His past medical history included asthma. He had no significant family history. He had a smoking history of approximately five pack-years and did not consume alcohol. He was not taking any regular medications.

**Figure 1:**
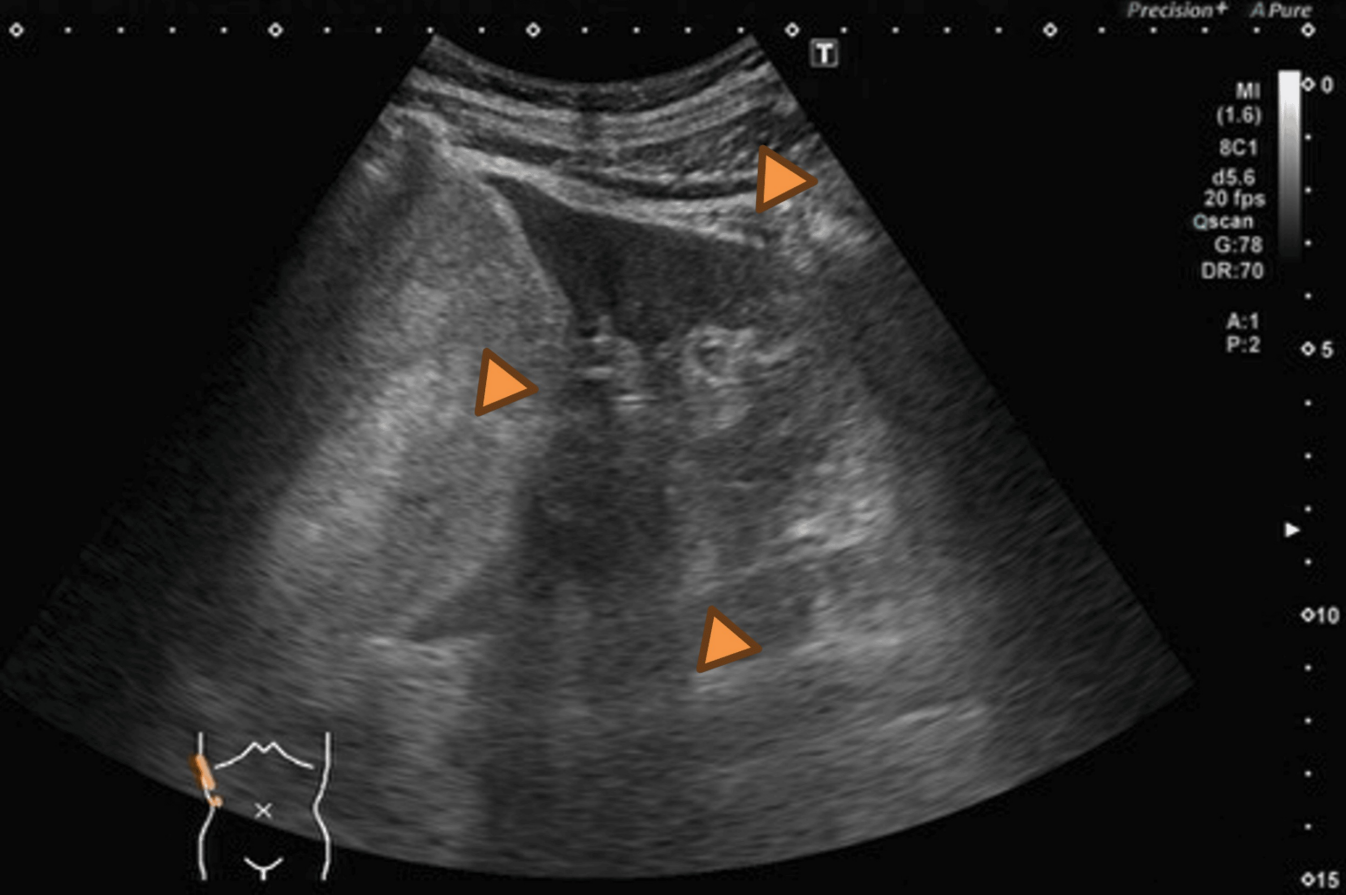
Abdominal ultrasonography findings Ultrasonography demonstrates a heterogeneous mass with mixed echogenic components and partially hyperechoic areas

**Figure 2:**
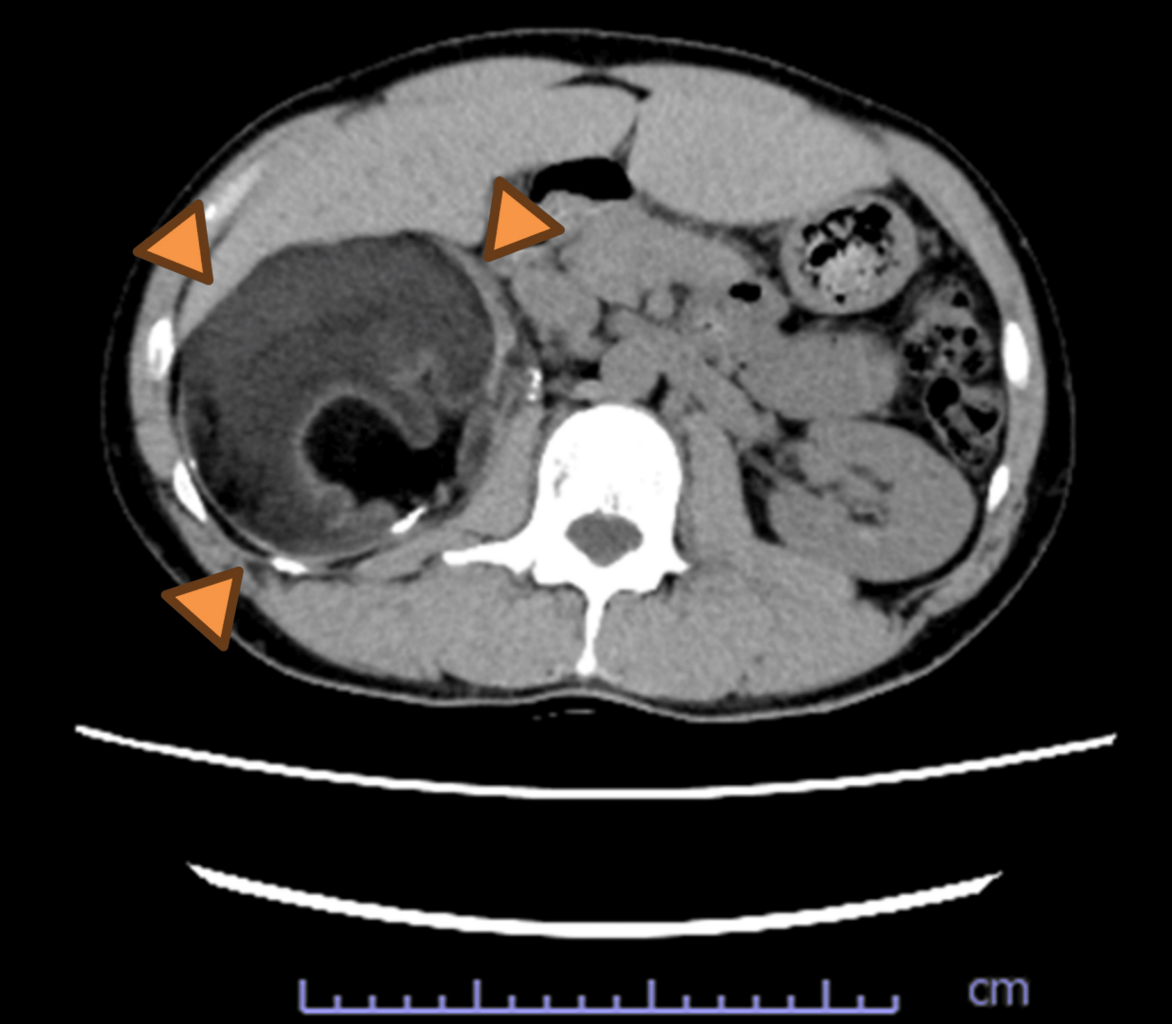
Abdominal CT findings CT shows a fat-containing mass measuring approximately 10 cm in diameter, located at the upper pole of the right kidney CT: computed tomography

Abdominal ultrasonography revealed a mass approximately 10 cm in diameter located near the upper pole of the right kidney (Figure 1). Laboratory findings were within normal limits (Table 1). The mildly elevated potassium level (5.4 mmol/L) was considered clinically insignificant and required no specific intervention. Non-contrast CT demonstrated a fat-containing mass, and renal angiomyolipoma was initially suspected (Figure 2). The patient was therefore referred to our department for possible transarterial embolization.

**Table 1:** Initial laboratory findings

Test	Result	Unit	Reference range
White blood cell count	4.57	x10^3^/µL	3.30–8.60
Hemoglobin	14.6	g/dL	13.7–16.8
Platelet count	320	x10^3^/µL	158–348
C-reactive protein	0.1	mg/dL	≤0.14
Aspartate aminotransferase	15	U/L	13–30
Alanine aminotransferase	12	U/L	10–42
Alkaline phosphatase	82	U/L	38–113
Lactate dehydrogenase	196	U/L	124–222
Total bilirubin	0.5	mg/dL	0.4–1.5
Blood urea nitrogen	16.5	mg/dL	8.0–20.0
Creatinine	0.75	mg/dL	0.65–1.07
Sodium	140	mmol/L	138–145
Potassium	5.4	mmol/L	3.6–4.8
Albumin	4.2	g/dL	4.1–5.1

**Figure 3:**
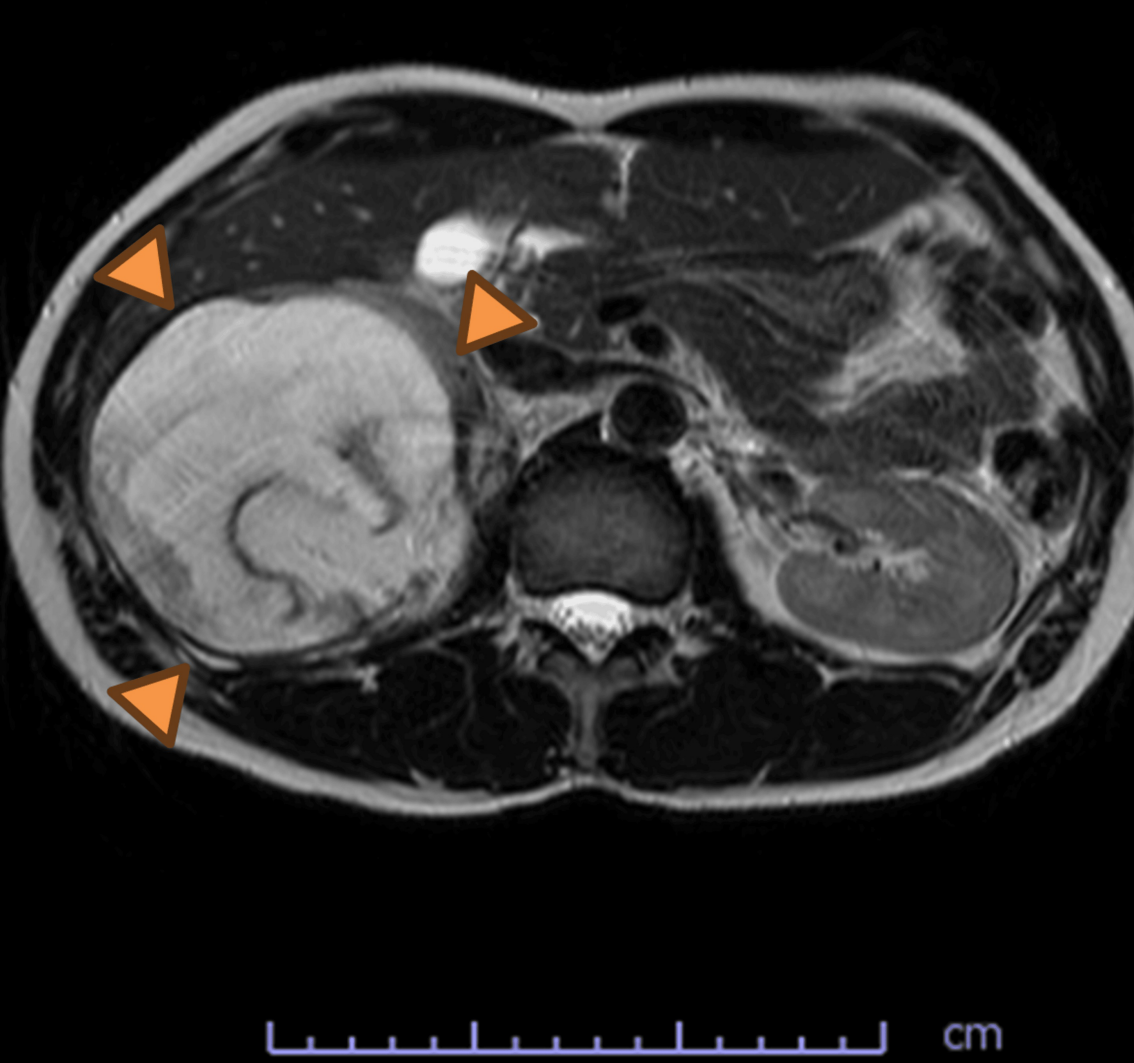
Abdominal MRI (T2-weighted image) findings MRI demonstrates a heterogeneous mass with internal septations and fat components MRI: magnetic resonance imaging

MRI revealed a heterogeneous mass containing fat components with internal septations and calcifications, raising suspicion for other retroperitoneal tumors, including liposarcoma (Figure 3). Although liposarcoma was considered in the differential diagnosis, the presence of mixed cystic and solid components with calcification suggested a teratomatous lesion rather than a purely adipocytic tumor. Because imaging findings alone could not reliably differentiate between benign and malignant disease, a tissue diagnosis was deemed necessary to determine the optimal treatment strategy. After incidental detection on routine ultrasonography, the patient was referred to our department within three weeks. Further imaging and percutaneous biopsy were performed, and surgical resection was carried out approximately three months after the initial detection.

After obtaining informed consent, an ultrasound-guided percutaneous core needle biopsy was performed using an 18-gauge needle, and multiple tissue cores were obtained without complications.

Histopathological examination of the biopsy specimen revealed keratinizing squamous epithelium, skin appendages, adipose tissue, and smooth muscle tissue, findings consistent with a mature teratoma. Serum tumor markers were within normal limits, with no elevation suggestive of a malignant germ cell tumor (Table 2).

**Table 2:** Serum tumor marker levels

Test	Result	Unit	Reference range
Alpha-fetoprotein (AFP)	2.57	ng/mL	0.89–8.78
Beta-human chorionic gonadotropin (β-hCG)	2.3	mIU/mL	<5.0
Soluble interleukin-2 receptor	431	U/mL	157–474

To evaluate for a possible testicular primary tumor or a burned-out testicular tumor with retroperitoneal metastasis, physical examination, testicular ultrasonography, and pelvic MRI were performed, all of which revealed no abnormalities in the testes. CT of the trunk demonstrated no evidence of distant metastasis. Based on these findings, the tumor was diagnosed as a primary retroperitoneal mature teratoma.

Surgical resection was planned. The operation was initially attempted using a retroperitoneoscopic approach. However, intraoperative findings revealed that the tumor was strongly adherent to both the upper pole of the right kidney and the liver, making laparoscopic dissection unsafe. Therefore, conversion to open surgery was performed to ensure complete resection and avoid potential complications. Because of the firm adhesion between the tumor and the right kidney, complete separation was considered unfeasible without risking incomplete resection, and the tumor was removed en bloc with the right kidney.

**Figure 4:**
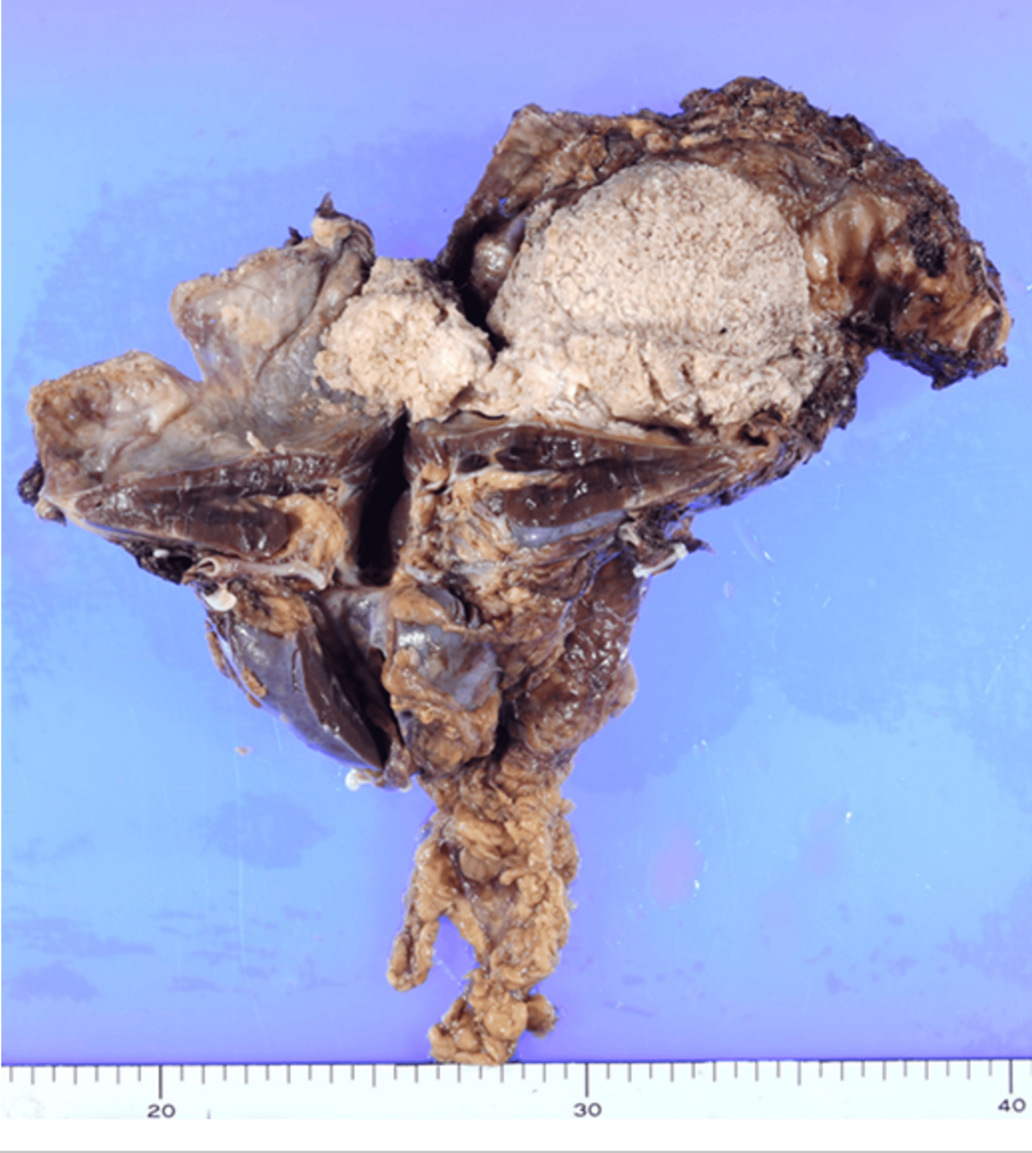
Resected specimen Resected specimen shows a cystic tumor containing hair-like material

**Figure 5:**
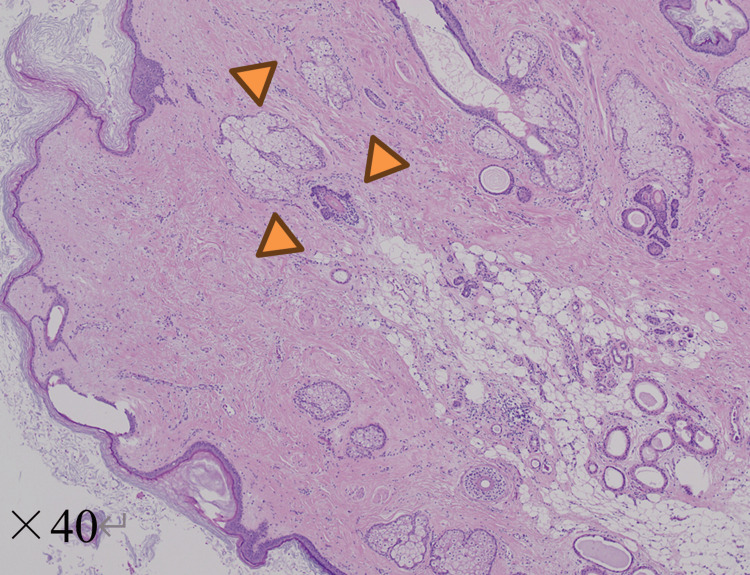
Histopathological findings Hematoxylin and eosin staining shows keratinized squamous epithelium and skin appendages, consistent with a mature cystic teratoma

The resected specimen showed a cystic tumor containing hair (Figure 4). Histopathological examination revealed keratinizing squamous epithelium, skin appendages, and adipose tissue, representing differentiation from multiple germ layers and confirming the diagnosis of a mature cystic teratoma; there were no immature components or malignant features (Figure 5). The heterogeneous components observed on imaging corresponded to multiple differentiated tissue elements identified on histopathological examination.

The postoperative course was uneventful, and the patient has remained free of recurrence for 18 months after surgery.

## Discussion

Fat-containing retroperitoneal tumors encompass a broad differential diagnosis, including renal angiomyolipoma, lipoma, and liposarcoma. Mature teratomas may demonstrate characteristic imaging findings such as fat components, calcifications, tooth-like structures, and cystic changes on CT or MRI [3]. However, differentiation from other retroperitoneal tumors can sometimes be difficult based solely on imaging findings [4,5]. In the present case, the tumor contained fat components and was initially suspected to be a renal angiomyolipoma on CT. However, additional imaging findings on MRI raised suspicion for other retroperitoneal tumors, including liposarcoma, making a definitive diagnosis difficult. Retroperitoneal teratomas are most commonly diagnosed in childhood, whereas adult cases are relatively rare [2]. As a result, mature teratoma is not always included in the differential diagnosis of retroperitoneal tumors in adult patients. Awareness of this entity is therefore important when evaluating fat-containing retroperitoneal masses.

Percutaneous needle biopsy for retroperitoneal tumors has historically been controversial because of concerns regarding tumor seeding along the biopsy tract in malignant tumors. However, several recent studies have suggested that the risk of tumor seeding is low and does not significantly affect local recurrence or overall survival [6,7]. When imaging findings are inconclusive, percutaneous biopsy can therefore be useful for establishing a diagnosis and determining an appropriate treatment strategy; however, its limitations, including the potential for sampling error, should also be recognized. Some recent case reports have also described the use of percutaneous biopsy for retroperitoneal teratomas, demonstrating its utility in establishing a preoperative diagnosis when imaging findings are inconclusive [8,9]. These reports support the role of biopsy as a valuable diagnostic tool in selected cases.

In the present case, the treatment strategy differed substantially depending on the diagnosis. Liposarcoma would have required a wider surgical resection with oncologic margins, whereas angiomyolipoma could potentially have been managed with transarterial embolization. Percutaneous biopsy enabled a definitive preoperative diagnosis, which facilitated appropriate surgical planning. In patients with retroperitoneal germ cell tumors, evaluation for a testicular primary tumor is essential because primary retroperitoneal germ cell tumors are rare [10]. In addition, the possibility of a burned-out testicular tumor must be considered [11]. In the present case, physical examination, testicular ultrasonography, and pelvic MRI revealed no abnormalities in the testes, and serum tumor markers, including alpha-fetoprotein (AFP), human chorionic gonadotropin (hCG), and lactate dehydrogenase (LDH), were within normal limits. These findings supported the diagnosis of a primary retroperitoneal mature teratoma.

Complete surgical resection is considered the treatment of choice for mature teratomas. Malignant transformation arising from squamous components has been reported in approximately 25% of adult cases, making complete resection particularly important [1]. While kidney-sparing approaches may be considered in selected cases, en bloc resection including adjacent organs, particularly the kidney, is often required when there is strong adhesion or difficulty in separating the tumor from surrounding structures [12]. In the present case, the tumor was strongly adherent to the upper pole of the right kidney, and en bloc resection with the right kidney was performed to ensure complete removal and minimize the risk of local recurrence.

## Conclusions

We reported a rare case of an adult retroperitoneal mature cystic teratoma arising in the perirenal region. Mature teratomas should be considered in the differential diagnosis of fat-containing retroperitoneal masses in adults. In this case, percutaneous needle biopsy enabled a definitive preoperative diagnosis when imaging findings were inconclusive and contributed to appropriate surgical planning. Percutaneous biopsy may therefore be a useful diagnostic tool in selected patients with complex retroperitoneal tumors.
